# A novel homozygous mutation in the glycerol-3-phosphate dehydrogenase 1 gene in a Chinese patient with transient infantile hypertriglyceridemia: a case report

**DOI:** 10.1186/s12876-018-0827-6

**Published:** 2018-06-25

**Authors:** Jia-Qi Li, Xin-Bao Xie, Jia-Yan Feng, Lian Chen, Kuerbanjiang Abuduxikuer, Yi Lu, Yu-Chuan Li, Jian-She Wang

**Affiliations:** 1 Department of Pediatrics, Jinshan Hospital of Fudan University, 1508 Longhang Road, Jinshan District, Shanghai, 201508 China; 2 The Center for Pediatric Liver Disease, Children’s Hospital of Fudan University, 399 Wanyuan Road, Minhang District, Shanghai, 201102 China; 3 Department of Pathology, Children’s Hospital of Fudan University, 399 Wanyuan Road, Minhang District, Shanghai, 201102 China

**Keywords:** Hypertriglyceridemia, *GPD1*, hepatomegaly, hepatic steatosis, HTGTI

## Abstract

**Background:**

Transient infantile hypertriglyceridemia (HTGTI) is an autosomal recessive disorder caused by mutations in the glycerol-3-phosphate dehydrogenase 1 (*GPD1*) gene.

**Case presentation:**

We report a case of HTGTI in a Chinese female infant. She presented with hepatomegaly, hypertriglyceridemia, moderately elevated transaminases, and hepatic steatosis at 3.5 months of age. A novel mutation c.523C>T, p. (Q175*) was identified in *GPD1.* The patient was a homozygote and her parents were heterozygous for the mutation. Ultrastructural study showed intrahepatocytic lipid droplets.

**Conclusions:**

This is the first reported case of HTGTI in Chinese, expanding the worldwide distribution of HTGTI and the mutation spectrum of *GPD1*.

**Electronic supplementary material:**

The online version of this article (10.1186/s12876-018-0827-6) contains supplementary material, which is available to authorized users.

## Background

The glycerol-3-phosphate dehydrogenase 1 (*GPD1*; MIM 138420) gene, which is mapped to chromosome 12q12-q13, encodes cytoplasmic NAD-dependent GPD1 that is crucial in both carbohydrate and lipid metabolism by catalyzing the reversible redox reaction of dihydroxyacetone phosphate (DHAP) and reduced nicotine adenine dinucleotide (NADH) to glycerol-3-phosphate (G3P) and NAD^+^ [1, 2]. Under physiological conditions, the reaction strongly favors the formation of G3P [2]. Glycerol-3-phosphate dehydrogenase 2 (*GPD2*; MIM 138430) is located on the outer surface of the inner mitochondrial membrane and catalyzes the unidirectional reaction of G3P and flavin adenine dinucleotide (FAD) to DHAP and FADH_2_ [2]. Together with a mitochondrial GPD2, GPD1 forms the G3P shuttle mainly in the brain and skeletal muscle of mammals, which transfers reducing equivalents from the cytosol to the mitochondria [3]. The human GPD1 is organized into two distinct domains, the *N*-terminal eight-stranded β-sheet sandwich domain (from residues 3–190) and the *C*-terminal helical substrate-binding (from residues 193–349) domain [2]. NAD^+^ binds to GPD1 at the periphery of its β-sheet core (10-GSGNWG-15) [2].

*GPD1* mutations were first identified by Basel-Vanagaite *et al.* as the cause of transient infantile hypertriglyceridemia (HTGTI; OMIM 614480) in 10 individuals from four consanguineous Israeli Arab families carrying a homozygous founder mutation c.361-1G>C [3]. HTGTI manifests as early onset hepatomegaly, hypertriglyceridemia, moderately elevated transaminases, hepatic steatosis, and hepatic fibrosis [3]. Recently, Li *et al*. reported biallelic mutations in *GPD1* gene of a Chinese boy who presented with a different phenotype comprising obesity, insulin resistance, fatty liver, and short stature [4]. To date, only four reports have described 16 patients harboring homozygous or compound heterozygous mutations in the *GPD1* gene [3–6]. Here, we describe a *Han* Chinese patient with a novel *GPD1* homozygous mutation who presented with hepatomegaly, elevated transaminases, hypertriglyceridemia, and fatty liver in infancy.

## Case presentation

This study was approved by the Research Ethics Committee of Children’s Hospital of Fudan University and was conducted under the Declaration of Helsinki ethical principles for medical research involving human subjects. Informed consent was obtained from the child’s parents.

### Patient

The female patient was the second child of a non-consanguineous *Han* Chinese couple. The mother previously had four episodes of spontaneous abortion during the first trimester due to lack of mixed lymphocyte reaction blocking antibodies. Through paternal lymphocyte immunotherapy, the mother successfully gave birth to the proband and her older brother. Fetal ultrasound scans of the proband showed fetal liver was normal. She was born at the gestational age of 39 weeks by cesarean section, with a birth weight of 3.15 kg. She presented at the age of 3 months and 14 days with bronchopneumonia, during the course of which elevated alanine aminotransferase (101 U/L, normal: 0-40 U/L) and aspartate aminotransferase (135 U/L, normal: 0-40 U/L) were noted. Initial abdominal ultrasound revealed hepatomegaly (the liver enlarged 2 cm below the costal margin). As the elevated liver enzymes and hepatomegaly persisted after recovery of bronchopneumonia, the child was transferred to another hospital for further treatment. Fasting hypertriglyceridemia was noted at presentation and throughout the follow-up period (range 4.39-10.94 mmol/L, normal: 0.56-1.7 mmol/L). Gamma-glutamyltranspeptidase and total bile acids were 265 IU/L (normal: 7-50 IU/L) and 18.2 μmol/L (normal: 0-10 μmol/L), respectively. However, total cholesterol, lipoprotein, bilirubin, alpha-fetoprotein and synthetic liver function (coagulation studies and albumin) were within normal limits. Abdominal CT scan showed a severely enlarged liver accompanied with fatty change, which had diffusely decreased density. Viral serological markers (hepatotrophic viruses, Epstein-Barr virus, cytomegalovirus, and HIV), toxoplasma, thyroid function tests, creatine phosphokinase, glucose, ceruloplasmin, blood routine tests, urine routine tests, blood lactate, blood ammonia, carbonyldiamide, creatinine, uric acid, urine organic acids, autoimmune serology, immunoglobulin levels, and echocardiogram were unremarkable. At this point, due to the combination of hypertriglyceridemia, hepatomegaly, and elevated liver transaminases, the working diagnosis was inclined to inherited metabolic liver disease. Thus, the whole blood samples from the family were sent to Precision MD (Beijing, China) for panel sequencing to screen 4503 genes using the customized xGen Inherited Diseases Panel. She took “Compound Glycyrrhizin” during this hospitalization. However, the levels of triglyceride and liver enzymes were not decreased. She was referred to the center for pediatric liver diseases in our hospital at 6.5 months of age for percutaneous liver biopsy. Since then, she did not receive any treatment. At the last evaluation at the age of 1 year and 3 months, the child still presented with hepatomegaly, hypertriglyceridemia, and moderately elevated transaminases, while maintaining a normal growth and psychomotor development. The detailed clinical characteristics of the proband are shown in Table 1. The patient’s parents and older brother were asymptomatic. The liver functions of the mother and older brother were normal. However, alanine aminotransferase (59 IU/L, normal: 0-40 IU/L), gamma-glutamyl transpeptidase (62 IU/L, normal: 7-50 IU/L), total cholesterol (5.87 mmol/L, normal: 3.1-5.2 mmol/L) and triglyceride (3.5 mmol/L, normal: 0.56-1.7 mmol/L) were mildly elevated in the father. In addition, the father was morbidly obese (body mass index 31.3 kg/m^2^, normal: 18.5-23.9 kg/m^2^).

**Table 1:** Clinical and laboratory findings of the patient

Personal history	
Presenting age (months)	3.5
Age at last following up (months)	15
Gravidity (G) and Parity (P)	G6P2
Gestation (weeks)	39
Birth weight (g)	3150
Birth history	cesarean section
Physical examination at 6.5 months old	
Head circumference (cm)	42.0
Chest circumference (cm)	43.0
Height (cm)	63.0
Weight (kg)	6.8
Temperature (°C)	37.0
Heart rate (beats per minute)	126
Respiration rate (times per minute)	28
Hepatomegaly	Yes
Splenomegaly	No
Biochemical examination	
Albumin (g/L)	45.1-50.8
Alanine aminotransferase (IU/L)	68-110
Aspartate aminotransferase (IU/L)	108-186
Gammaglutamyl-transpeptidase (IU/L)	233-482
Direct bilirubin (umol/L)	1.0-2.3
Total bilirubin (umol/L)	2.4-9.4
Total bile acid (umol/L)	9.0-19.8
Alpha fetal protein (ng/mL)	53
Triglyceride (mmol/L)	4.18-10.94
Total cholesterol (mmol/L)	2.36-4.35
Glucose (mmol/L)	4.2-5.7
Coagulation test	
D-dimer (mg/L)	0.27-1.18
Activated partial thromboplastin time (s)	25.0-33.3
Thrombin time (s)	19.4-19.9
Prothrombin time (s)	11.0-13.3
Fibrinogen (g/L)	1.84-1.93
Blood routine examination	
Hemoglobin (g/L)	95.0-101.0
Red blood cell count (per liter)	4.91-5.24 × 10^12^
White blood cell count (per liter)	7.7-11 × 10^9^
Lymphocytes (%)	65.0-75.0
Neutrophils (%)	16.8-22.0
Platelets count (per liter)	568-782 × 10^9^

### Genetic analysis

Written informed consent was obtained prior to the collection of peripheral blood from the proband and her family members. All the blood samples were sent to Precision MD (Beijing, China) for sequencing. Genomic DNA of all the family members was extracted from their peripheral blood leukocytes according to the manufacturer's standard procedure using the QIAamp DNA Blood Midi Kit (Qiagen, Hilden, Germany). Genomic DNA of the proband underwent panel sequencing to screen 4503 genes using the customized xGen Inherited Diseases Panel. DNA was fragmented by Covaris LE220 (Massachusetts, USA) to generate paired-end library (200–250 bp). Targets were captured using xGen Lockdown Probes (Precision MD, Beijing, China). The captured libraries were sequenced using the Illumina hiseq2500 Analyzers following the manufacturer’s instructions (Illumina, San Diego, USA). The reads were mapped to human genome reference (hg19) using Burrows-Wheeler Aligner (http://bio-bwa.sourceforge.net/). Single-nucleotide variants and indels were called with SOAPsnp (The Beijing Genomics Institute in-house software) and Samtools (http://samtools.sourceforge.net/). All single-nucleotide variants and indels were filtered via multiple databases. Pathogenic variants were assessed under the protocol issued by the American College of Medical Genetics and Genomics [7]. Mutations were verified in the proband and her family members by Sanger sequencing.

### Molecular findings

A homozygous nonsense mutation c.523C>T, p. (Q175*) in *GPD1* (NM_005276.3) was identified in the proband. Her parents and older brother were heterozygous for the mutation, consistent with a recessive inheritance (Additional file 1). This variant introduces a stop codon at codon 175, which is predicted to result in nonsense mediated mRNA decay by MutationTaster (http://www.mutationtaster.org/) [8]. The variant was novel and not recorded in Exome Aggregation Consortium Server (http://exac.broadinstitute.org/), NHLBI Exome Sequencing Project (http://evs.gs.washington.edu/EVS/) and Thousand Genomes Project (http://www.1000genomes.org/home).

### Pathological findings

**Fig. 1:**
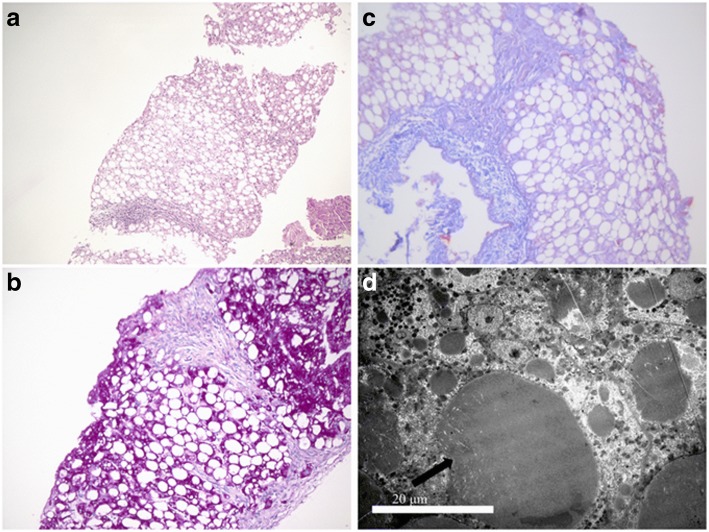
The pathological results of the proband. **a** Marked macro- and microvacuoles were observed (Hematoxylin and Eosin staining; original magnification, ×100). **b** Periodic acid Schiff staining also showed macro- and microvacuoles in hepatocytes (×200). **c** Mild fibrosis was presented in portal tracts (Masson staining, ×200). **d** The arrow showed fats were in the hepatocytes

Liver biopsy was performed at 6.5 months of age. Light microscopy revealed marked micro- and macro- vacuoles in hepatocytes on hematoxylin and eosin stained sections, indicating severe hepatic steatosis (Fig. 1a). Mild lymphocytic inflammatory infiltrates in the portal tracts were also present. Periodic acid Schiff staining showed macro- and micro- vacuoles in hepatocytes (Fig. 1b). Masson staining revealed mild portal fibrosis (Fig. 1c). Immunostaining for cytokeratin 7 and 19 demonstrated slight proliferation of the bile ducts along the ductal plate. Iron, HBsAg, HBcAg and Epstein-Barr virus were negative. Electron microscopy revealed macro- and micro fat and glycogen in the cytoplasm of hepatocytes, indicating severe liver steatosis (Fig. 1d). Increased myeloid body, abundant collagen fibers and mild dilation of bile canaliculi were also observed.

## Discussion and conclusions

HTGTI is a rare autosomal recessive disorder that has been described in a total of 15 affected individuals in three studies [3, 5, 6]. The shared features of these patients include infantile hypertriglyceridemia, elevated liver enzymes, hepatomegaly, liver steatosis and fibrosis. Additional uncommon phenotypes include fasting hypoglycemia and kidney disease. We report the first *Han* Chinese patient with HTGTI, and the patient carried a novel homozygous nonsense mutation in the *GPD1* gene, which is the causative variation. Our patient clinically resembled other reported children with HTGTI: She presented with elevated liver transaminases, hypertriglyceridemia, marked hepatomegaly, and hepatic steatosis since early infancy. Electron microscopy was only undertaken in one of 15 previous affected patients, revealing interhepatocytic, not intrahepatocytic, vesicles (most likely fat) [6]. By contrast, electron microscopy showed intrahepatocytic, not interhepatocytic, lipid droplets in our patient; however, the significance of this difference is unclear. Although our patient was not given treatment since 6.5 months old, the levels of triglyceride and liver enzymes decreased at the last evaluation at the age of 1 year and 3 months. The heterozygote (carrier) parents and older brother of our reported patient were asymptomatic. The mildly elevated liver functions of the father may be due to his high body mass index.

Recently, Li *et al*. reported biallelic mutations (c.220-2A>G and c.820G>A) in *GPD1* gene of a 13-year, 8-month-old Chinese boy who presented with obesity, insulin resistance, fatty liver, dermal abnormalities (facial acne, acanthosis nigricans, and hirsutism), short stature, elevated dehydroepiandrosterone sulfate and lipoprotein-α levels, which were different from HTGTI phenotype [4]. Clinical features and molecular genetics of individuals with biallelic mutations in *GPD1* gene were shown in Table 2. Li *et al*. also suggested that the *GPD1* gene should be considered as the short stature causing gene [4]. However, it is uncertain that the different phenotype of the adolescent patient was due to phenotypic heterogeneity or having an unrecognized second disorder.

**Table 2:** Clinical features and molecular genetics of individuals with biallelic mutations in *GPD1* gene

Patient	F1-IV1	F1-IV2	F1-IV4	F2-II6	F2-III1	F3-V1	F3-V2	F3-V3	F4-II4	F4-III3	11	A	B	C	D	16	17
Gender	M	M	M	M	F	F	F	F	M	M	F	M	F	M	M	M	F
Descent	Israeli Arab										Caucasian	Arab-Muslim	NA	Italian	Italian	Chinese	Chinese
Consanguinity	Yes	Yes	Yes	Yes	Yes	Yes	Yes	Yes	Yes	Yes	NA	Yes	No	Yes	NA	No	No
Term birth	NA	NA	No	NA	Yes	Yes	Yes	Yes	No	No	Yes	NA	Yes	Yes	NA	Yes	Yes
Birth weight (g)	3,000	NA	2,180	3,395	2,570	3,000	2,430	2,190	1,950	2,160	NA	NA	3,500	3,640	NA	2900	3,150
Presenting age (m)	1	1	4–6	Birth	6	2.5	7	7	9	3.5	Birth	10	12	5	24	84	3.5
Age at last test (y)	13.7	9.9	11.9	23	2.9	4.3	1.3	1.3	12.5	12.5	1.5	4.5	4	7	31	NA	1.2
Initial TG (mg/dl)	6244	250	990	520	1208	349	258	330	225	566	839	170	1180	466	213	N	388
Last TG (mg/dl)	250	247	289	170	202	135	185	202	301	434	536	N	N	271	NA	NA	370
TG^a^(mg/dl)	10–150										50-130	40-150				NA	50-150
Initial CH (mg/dl)	420	109	164	101	109	66	95	99	106	149	197	155	260	N	NA	N	95
Last CH (mg/dl)	73	207	172	114	184	202	118	141	188	256	NA	NA	NA	NN	204	NA	168
CH^a^ (mg/dl)	<170										<170	120-200				NA	120-201
Elevated transaminases	Yes	Yes	Yes	Yes	Yes	Yes	Yes	Yes	Yes	Yes	Yes	Yes	Yes	Yes	Yes	No	Yes
Hepatomegaly	Yes	Yes	Yes	Yes	Yes	Yes	Yes	Yes	Yes	Yes	Yes	Yes	Yes	Yes	Yes	No	Yes
Splenomegaly	NA	NA	No	Yes	NA	No	Yes	Yes	No	No	NA	No	No	Yes	NA	No	No
Hepatic steatosis	Yes	Yes	Yes	Yes	Yes	Yes	Yes	Yes	Yes	Yes	Yes	Yes	Yes	Yes	Yes	Yes	Yes
Short stature	No	No	Yes	Yes	No	No	No	No	Yes	Yes	No	NA	NA	No	NA	Yes	No
Other findings	No	No	No	No	No	No	No	No	Horseshoe kidney; Transient hypotonia	Craniocerebral involvement	No	Fasting hypoglycemia	No	Kidney involvement	No	Obesity; Insulin resistance; Dermal abnormalities; EDL	No
Age of liver biopsy	NA	2.5y	4.5y	NA	NA	NA	NA	NA	NA	NA	5m	NA	1y	5m<Age<1y	NA	NA	6.5m
Light microscopy of liver	NA	S; FI; MI	S; FI; MI	NA	NA	NA	NA	NA	NA	NA	S	NA	S; FI	S; C; I	S; FI	NA	S; FI; MI
Electron microscopy of liver	NA	NA	NA	NA	NA	NA	NA	NA	NA	NA	NA	NA	Vesicles between the hepatocytes	NA	NA	NA	Lipid droplets in hepatocytes
Mutations (NM_005276)	c.361-1G>C; Homo										R229Q+ a long deletion	Arg269Gln; Homo	c.361- 1G > C; Homo	Cys214Arg; Homo	Cys214Arg; Homo	c.220-2A>G + Ala274Thr	Q175^a^; Homo
Reference	Basel-Vanagaite *et al*., 2012										Joshi *et al*., 2014	Dionisi-Vici *et al*., 2016				Li *et al*., 2017	This study

*TG* triglyceride, *CH* cholesterol, *S* steatosis, *FI* fibrosis, *MI* mild inflammation, *C* cirrhosis, *I* inflammation, *EDL* elevated dehydroepiandrosterone sulfate and lipoprotein-α levels, *NA* no available, *NN* near normal values, *N* normal, *Homo* homozygote, *F* femal, *M* male, *g* grams, *m* months, *y* years

^a^reference range

Hypertriglyceridemia is a hallmark of many disorders, including metabolic syndrome, diabetes, atherosclerosis, and obesity. Several genetic defects for hypertriglyceridemia have been identified, including mutations in *APOA5* (MIM 606368) [9], *LIPI* (MIM 609252) [10], *LPL* (MIM 609708) [11], *APOC2* (MIM 608083) [12], *LIPC* (MIM 151670) [13], *USF1* (MIM 191523) [14], *GPIHBP1* (MIM 612757) [15], and *LMF1* (MIM 611761) [16]. *GPD1* disease is one of the important molecular etiologies of primary hypertriglyceridemia with onset in infancy [3]. Basel-Vanagaite *et al.* confirmed that mutation of *GPD1* in HepG2 cells causes increased triglyceride synthesis and secretion [3]. However, the exact mechanism of hypertriglyceridemia in *GPD1* deficiency is unclear and remains to be further clarified. Notably, high triglyceride level is an independent risk factor for coronary artery disease and also associated with an increased risk of acute pancreatitis [17–19]. The long term effects of hypertriglyceridemia on patients with HTGTI is unknown, and it is necessary to pay attention to these patients in their long-term follow up.

All the *GPD1* deficiency patients had fatty liver on ultrasound or other imaging examinations. Light microscopy undertaken in 6 of sixteen previous affected patients showed steatosis with varying degree of fibrosis. Joshi M *et al.* proposed that fatty liver in *GPD1* disease may be due to acylation of excess DHAP [5]. The detailed mechanisms are not clear and further research is needed to shed light on them. Fatty liver with hepatomegaly can be caused by a spectrum of inherited metabolic liver diseases, such as glycogen storage disease, lipidosis, lysosomal diseases, and citrin deficiency. Liver biopsy and genetic analysis are both useful for differential diagnosis of HTGTI and those diseases. However, the definite diagnosis of HTGTI depends on genetic testing.

The natural history of HTGTI is not clear yet. Although most patients presented with liver fibrosis in early infancy, all affected individuals had a good prognosis. At the time of their last evaluation, the levels of triglyceride and liver enzymes were improved in most patients. In addition, the oldest patient (aged 31 years) was doing well [6]. Thus, liver transplantation is not recommended for HTGTI patients. Since triglyceride levels could be improved without specific therapy, we don’t recommend lipoprotein apheresis as routine therapy for these patients. But it could be an option for patients with severe hyperlipemia. We recommend to evaluate the growth and development and test liver function, total cholesterol, triglycerides, abdominal ultrasound, FibroScan and other abnormal index in the follow-up visit. In addition, it is necessary to pay attention to coronary artery disease and pancreatitis in their long-term follow up, which is associated with hypertriglyceridemia.

Previously, a total of 5 mutations in the *GPD1* gene have been identified in 15 patients with HTGTI. In this study, we reported a novel disease-causing mutation c.523C>T, which is predicted to result in nonsense mediated mRNA decay. Among the 16 patients carrying biallelic mutations in *GPD1*, 15 patients showed a homozygous mutation, while only 1 patient was compound heterozygotes. The identified variants comprised 1 nonsense (c.523C>T), 3 missense (c.686G>A, c.806G>A, and c.640T>C), 1 splice site variant (c.361-1G>C), and 1 deletion mutation (28.7 kb > the deletion size > 1.85 kb).

In conclusion, we reported a *Han* Chinese HTGTI patient with mutation of *GPD1*, having hypertriglyceridemia, hepatomegaly, mildly elevated transaminases, and hepatic steatosis. *GPD1* deficiency should also be considered in children and adolescents with hypertriglyceridemia and hepatic steatosis of unknown causes, and that the natural history of the condition is still unknown.

## Additional file

Additional file 1: Mutation analysis of the *GPD1* gene in the proband and her parents. The patient was a homozygote and her parents were heterozygous for the mutation. (DOCX 853 kb)

## Abbreviations

DHAP: Dihydroxyacetone phosphate
FAD: Flavin adenine dinucleotide
G3P: Glycerol-3-phosphate
GPD1: Glycerol-3-phosphate dehydrogenase 1
GPD2: Glycerol-3-phosphate dehydrogenase 2
HTGTI: Transient infantile hypertriglyceridemia
NADH: Reduced nicotine adenine dinucleotide
