# Supplementary material for: A novel homozygous mutation in the glycerol-3-phosphate dehydrogenase 1 gene in a Chinese patient with transient infantile hypertriglyceridemia: a case report
Source: BMC Gastroenterol. 2018 Jun 25;18:96. doi: 10.1186/s12876-018-0827-6 (PMC6020200; doi:10.1186/s12876-018-0827-6)
Supplement: Supplementary file 1 — Mutation analysis of the GPD1 gene in the proband and her parents. The patient was a homozygote and her parents were heterozygous for the mutation. (DOCX 853 kb) [file 12876_2018_827_MOESM1_ESM.docx]

**Additional file 1** Mutation analysis of the *GPD1* gene in the proband and her parents.


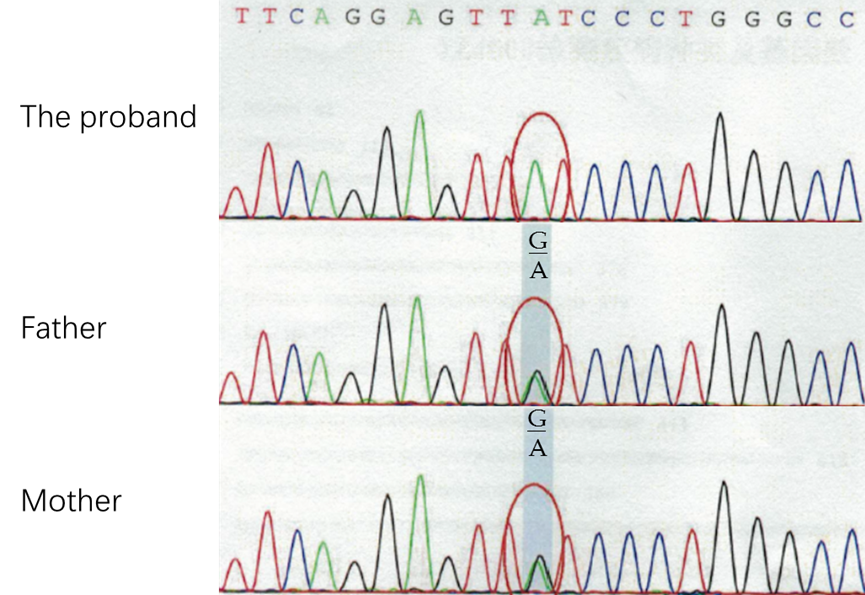


The patient was a homozygote and her parents were heterozygous for the mutation.
